# IPEX as a Consequence of Alternatively Spliced FOXP3

**DOI:** 10.3389/fped.2020.594375

**Published:** 2020-10-21

**Authors:** Reiner K. Mailer

**Affiliations:** Institute of Clinical Chemistry and Laboratory Medicine, University Medical Center Hamburg-Eppendorf, Hamburg, Germany

**Keywords:** Foxp3, isoform, alternative splicing, IPEX, CD4^+^ T cell

## Abstract

The transcription factor FOXP3 controls the immunosuppressive program in CD4^+^ T cells that is crucial for systemic immune regulation. Mutations of the single X-chromosomal *FOXP3* gene in male individuals cause the inherited autoimmune disease immune dysregulation, polyendocrinopathy, enteropathy, and X-linked (IPEX) syndrome. Insufficient gene expression and impaired function of mutant FOXP3 protein prevent the generation of anti-inflammatory regulatory T (Treg) cells and fail to inhibit autoreactive T cell responses. Diversification of FOXP3 functional properties is achieved through alternative splicing that leads to isoforms lacking exon 2 (FOXP3Δ2), exon 7 (FOXP3Δ7), or both (FOXP3Δ2Δ7) specifically in human CD4^+^ T cells. Several IPEX mutations targeting these exons or promoting their alternative splicing revealed that those truncated isoforms cannot compensate for the loss of the full-length isoform (FOXP3fl). In this review, IPEX mutations that change the FOXP3 isoform profile and the resulting consequences for the CD4^+^ T-cell phenotype are discussed.

## Introduction

Immune dysregulation, polyendocrinopathy, enteropathy, X-linked (IPEX) syndrome is a rare condition affecting less than one in a million newborns. Because patients with IPEX syndrome may develop a wide range of symptoms with varying severity, it has been estimated that cases remain often undiagnosed and the prevalence of the disease is higher than reported. Clinical presentation of IPEX syndrome include systemic autoimmune manifestations (dermatitis/eczema, autoimmune hemolytic anemia, atopy), enteropathy (severe diarrhea, food allergies), endocrinopathy (type 1 diabetes mellitus, thyroiditis), and a failure to thrive. The triad of dermatitis, diarrhea and diabetes in male infants is applicable as disease indicator and if left untreated IPEX syndrome becomes a life-threatening condition early in life. The disease is commonly inherited from the maternal lineage and is disseminated in an X chromosome-linked recessive pattern. Because fetal immunity develops already during the second trimester of gestation, intrauterine immune dysregulation has been linked to fetal death and repeated miscarriages may point toward an IPEX mutation in one of the maternal X chromosomes [reviewed in Bacchetta et al. (1)]. IPEX syndrome is a monogenic disease caused by the mutation of the *forkhead box P3* (*FOXP3*) gene, that encodes for the transcription factor FOXP3 (initially also named JM2 and Scurfin) (2, 3). Analysis of *FOXP3* mutations from patients and patients' mothers promptly confirm IPEX syndrome diagnosis and identify IPEX mutation carrier, respectively.

## IPEX Mutations Target FOXP3—The Indispensable Immune Regulator

Foxp3 gained intensive research interest since it was described in 2003 as the central lineage-defining marker for regulatory T (Treg) cells (4, 5) that maintains immunologic self-tolerance through the development of the immunosuppressive CD4^+^CD25^+^ T cell subset (6). Absence of Foxp3 in mice (7) and FOXP3 in humans (2, 8) cause fatal multiple organ autoimmunity termed scurfy and IPEX syndrome, respectively. In line with the pivotal role of the transcription factor retroviral transduction of murine Foxp3 (4, 5) and human FOXP3 (9) induces a Treg-cell phenotype and confers suppressive capacity on CD4^+^CD25^−^ T cells. FOXP3 broadly regulates the gene expression profile (10), which is related to transcriptional and epigenetic changes triggered by T-cell antigen receptor (TCR) stimulation (11, 12). Moreover, TCR signaling is necessary to maintain Treg-cell functions (13, 14). Importantly, *FOXP3* transcription is controlled by chromatin modifications at conserved non-coding sequences (CNS) (15). CNS3 located at *FOXP3 intron 1* (i.e., between exon 1 and 2) acts as a genetic switch that initiates gene expression through interactions with c-rel (15, 16) and atypical NF-κB inhibitor (17). Accordingly, targeting CNS3 chromatin accessibility with a specific ribonucleoprotein (dCas9—catalytically inactive variant of clustered regularly inter-spaced short palindromic repeats-associated protein 9) has been shown to promote FOXP3 expression in Jurkat cells (18), whereas endogenous long non-coding RNA (*FLICR*—*Foxp3* long intergenic non-coding RNA) induces a FOXP3^low^ T-cell subset (19). Although high FOXP3 expression levels are commonly attributed to CD4^+^CD25^high^ Treg cells, FOXP3 is also induced by TCR stimulation in naive CD4^+^CD25^−^ T cells and persists for several days in activated CD4^+^ T cells (20). Notably, activation-induced FOXP3 expression seems to depend on the CNS3 element, because generation of transgenic mice with a bacterial artificial chromosome that encodes for a reporter protein inserted into the human *FOXP3* gene (consisting of >50 kb each side of the start codon, including CNS1 and CNS2, but not CNS3) demonstrated that TCR stimulation alone (i.e., without transforming growth factor-β activation) does not induce transgenic FOXP3 expression in murine CD4^+^ T cells (21).

Moreover, in the absence of antigenic stimulation, FOXP3 expression is promoted in CD4^+^CD25^−^ T cells through STAT5-signaling cytokines IL-2, IL-7, and IL-15 (22) and short term exposure with these cytokines has been reported to reveal higher portions of “latent” Treg cells among human peripheral blood mononuclear cells (23). Although addition of cytokines increased the number of Treg cells for autologous transfer trials, this treatment may increase plasticity of Treg cells and reduce susceptibility of reactive T cells toward Treg-mediated suppression (24–26). In contrast to peripheral blood, cytokine treatment does not induce FOXP3 expression in CD4^+^ T cells obtained from lymph nodes (23); thus consequences for T-cell responses in disease settings remain unknown. Flow cytometry analysis of CD4^+^ T cells from peripheral blood demonstrated that FOXP3 expression varies up to 40-fold between individual cells. Strength and duration of FOXP3 induction may therefore explain discrepancies in the suppressive capacity of T cells with ectopically expressed or activation- and cytokine-induced FOXP3 (20, 22, 23, 27–30). *In vivo*, CD4^+^ T cells with activation-induced FOXP3 expression should accumulate at active sites of inflammation. Indeed CD4^+^CD25^−^FOXP3^+^ and CD4^+^CD45RA^−^FOXP3^low^ populations increase in inflamed tissue of patients with autoimmune diseases and this has been attributed to self-reactive non-Treg cell responses (31–34). The notion that CD4^+^ T-cell subsets in different tissues express distinct patterns of CD25 and other Treg-cell markers in both mice and men (35–38), adds even more complexity to the task to safely distinguish FOXP3^+^ Treg from FOXP3^+^ non-Treg cells. One important target for FOXP3-mediated transcriptional repression is the promoter of the IL-7 receptor (CD127). Accordingly, the most suppressive human Treg-cell population has been identified to express a CD4^+^CD25^+^CD127^low^ phenotype (39, 40). However, the corresponding CD4^+^CD25^−^CD127^+^ conventional T (Tcon) cells rapidly decrease CD127 upon IL-7 signaling and TCR stimulation (41), whereas CD127 expression has been reported to increase in activated Treg cells in mice (42).

Taken together, FOXP3 induction in human CD4^+^ T cells prevents unambiguous identification of immunosuppressive and immunoreactive T-cell subsets that challenges assessment of T-cell responses in autoimmunity [reviewed in Scheinecker et al. (43) and Buckner (44)]. So far it remains elusive, why murine Tcon cells do not induce Foxp3 upon stimulation likewise to human Tcon cells, because both species have the CNS3 as well as a Treg-associated gene expression profile similar to stimulated Tcon cells in common (45, 46). In human Tcon cells, activation-induced FOXP3 intrinsically restricts T-cell proliferation and differentiation (47–49) and protects stimulated Tcon cells from premature restimulation-induced cell death (50). Further studies are required to investigate susceptibility, spatio-temporal expression and isoform-specific functions of activation-induced FOXP3 in stimulated Tcon cells.

## FOXP3 Isoform Expression

Alternative splicing of human FOXP3 leads to isoforms lacking exon 2 (FOXP3Δ2), exon 7 (FOXP3Δ7), or both exon 2 and 7 (FOXP3Δ2Δ7) (51). Several studies revealed that FOXP3 splice variants confer partly overlapping but also isoform-specific functions (Table 1). FOXP3Δ2 induces immunosuppressive phenotype and function cooperatively with FOXP3fl in human and murine CD4^+^ T cells (27, 52). Although FOXP3Δ2 lacks the LXXLL interaction motif for binding retinoid orphan receptor-γt (the transcription factor ROR-γt that promotes the differentiation of pro-inflammatory Th17 cells) (53), FOXP3Δ2 has not been associated with Th17-related disease (54–56). Moreover, exclusion of exon 2 in activation-induced FOXP3 through splice-shifting antisense oligonucleotides significantly decreases expression of IL-17A, but not IL-2, and IFN-γ, demonstrating that FOXP3Δ2 facilitates restraint on T cell responses and effectively prevents rather than promotes Th17-cell differentiation (55). Consistently with immunosuppressive function of FOXP3Δ2, decreasing ratios of FOXP3fl/FOXP3Δ2 has been interpreted as functional gain in long-term activated Treg cells (57), whereas a functionally exhausted Treg-cell status has been proposed for this phenotype in autoimmunity (58, 59). A recent study by the latter group showed that FOXP3 isoforms containing exon 2 increase stronger than FOXP3 isoforms lacking exon 2 in peripheral CD4^+^ T cells of patients suffering from a moderate stage of chronic obstructive pulmonary disease (60). Moreover, expression of the proliferation marker Ki-67 increases in cell populations stained with FOXP3 exon 2-specific antibodies compared to those stained with antibodies that detect all FOXP3 isoforms. This is in line with our results that TCR stimulation has an impact on the FOXP3fl/FOXP3Δ2 ratio (61). Detection of FOXP3 exon 2 in relation to total FOXP3 by specific antibodies revealed that alternative splicing shifted toward increased inclusion of exon 2 upon antigenic stimulation. Moreover, this activation-induced FOXP3 isoform pattern has been observed in immunostimulatory settings, such as coronary artery disease (61), inflammatory bowel disease (54) and myelodysplastic syndrome (62). The latter report also describes a prevalence for FOXP3fl expression during T cell development in the thymus. Consistently, we recently found a higher expression of FOXP3fl compared to FOXP3Δ2 in thymocytes and that FOXP3fl precedes alternative splicing of exon 2 in TCR-stimulated naive T cells (manuscript in preparation). The development of IPEX caused by *FOXP3 exon 2* mutations that abrogate expression of FOXP3fl but otherwise enable unchanged FOXP3Δ2 expression, highlights the importance of FOXP3fl for T cell homeostasis (see below).

**Table 1:** Characteristics of FOXP3 isoforms.

	**FOXP3 full length**	**Deletion of exon 2**	**Deletion of exon 7**
FOXP3 protein length	431 amino acids	−35 amino acids	−27 amino acids
Expression in peripheral Treg cells	50–30%	70–50%	1–3%
Immunosuppressive function	Yes	Yes	No
Blockade of IL-2 expression	Yes	Yes	No
Induction of CD4^+^CD25^+^C127^low^ phenotype	Yes	No	No
Supporting own expression	Yes	No	No
Induction by TCR stimulation	strong	weak	weak
Th17 differentiation	Inhibition	Inhibition	Enhancement

Additional splicing of *FOXP3 exon 7* generates the isoform FOXP3Δ2Δ7 in human CD4^+^ T cells. FOXP3Δ2Δ7 does not contribute to the suppressive phenotype and function of Treg cells in mice and men (52, 63). Exclusion of exon 7 is significantly decreased in activated Treg cells (55). However, IL-1β strongly induces alternative splicing of *FOXP3 exon 7* in Treg cells and expression of *FOXP3* lacking exon 7 is elevated in colon biopsies obtained from Crohn's disease patients. Moreover, antisense oligonucleotide-mediated splicing of *FOXP3 exon 7* in naive CD4^+^ T cells promotes the differentiation of pro-inflammatory Th17 cells (55). Mostly described for inflamed tissues, phenotypically mixed CD4^+^FOXP3^+^IL-17A^+^/ROR-γt^+^ T cells have been identified [reviewed in Du et al. (64)]. However, the extent of *FOXP3 exon 7* splicing and the threshold that sufficiently drives Treg-cell plasticity in these cells is currently unknown. FOXP3 exon 7 is part of the protein's leucine zipper domain regulating the composition and stability of the multi-protein transcriptional repressor complex (65, 66); nevertheless about one third of FOXP3 target genes are similarly expressed in stimulated CD4^+^ T cells in the absence of FOXP3 exon 7 (67) and FOXP3Δ2Δ7 inhibits FOXP3fl in a dominant negative manner (52). Thus, both complementary and competing functions are carried out by FOXP3 isoforms with alternative splicing of exon 7. IPEX mutations within *FOXP3 exon 7* and particularly intronic mutations that mediate excessive splicing of *FOXP3 exon 7* demonstrate that endogenous FOXP3Δ2Δ7 or aberrant FOXP3Δ7/FOXP3Δ2Δ7 expression cannot compensate for the loss of FOXP3 isoforms with exon 7 (see below).

Taken together, the analysis of FOXP3 isoforms can elucidate impaired tolerance induction and exaggerated T-cell responses in autoimmunity and chronic diseases. In healthy conditions, FOXP3 isoforms are co-expressed in CD4^+^ T cells but may differ in their respective ratio depending on TCR stimulation and cytokine activation. The assessment of isoform-specific functions is challenging because modulation of human CD4^+^ T cells (e.g., via transfection or transduction) induces FOXP3 expression. However, IPEX mutations that target alternative splicing or that cause nonsense-mediated decay of frameshifted mRNA facilitate the analysis of naive CD4^+^ T cells with limited FOXP3 isoform variation. Thus, *FOXP3* sequencing in IPEX patients and female relatives together with flow cytometric analysis of CD4^+^CD25^+^CD127^−^ T cells in pediatric case reports contribute to our understanding of FOXP3 functions and Treg-cell development.

## Differential Effect of IPEX Mutations That Target the FOXP3 Isoform Ratio

Since the monogenic primary immunodeficiency IPEX syndrome was defined more than 170 distinct *FOXP3* mutations have been reported (68). These mutations likely represent severe cases with various clinical symptoms, whereas milder forms may go undetected. However, it has become clear that identical *FOXP3* mutations can cause different disease manifestation in IPEX patients (69). This reflects the stochastic nature of autoreactive responses [reviewed in Richards et al. (70)], compensatory mechanisms to suppress autoreactive T-cell responses [e.g., FOXP3-independent type 1 regulatory T cells (71)] and the balance of Treg- and Tcon-cell functions affected by the IPEX mutation (47, 48). Depending on disease severity, combinatorial therapy with immunosuppressive drugs (e.g., rapamycin, glucocorticoids, cyclosporine, tacrolimus) can alleviate autoimmunity in IPEX patients, whereas the only curative approach for IPEX syndrome is allogeneic hematopoietic stem cell transplantation. An analysis of currently known IPEX cases with *FOXP3* mutations that abrogate the correct expression of FOXP3 exon 2 (five frameshift mutations) and FOXP3 exon 7 (four deletion/missense mutations, seven splicing mutations) and the impact on clinical parameters and the corresponding T cell phenotype is shown (Table 2).

**Table 2:** Mutations that target FOXP3 isoform expression ratios cause IPEX.

	**cDNA**	**Protein**	**Immune dysregulation**	**Endocrinopathy**	**Enteropathy**	**Onset**	**Case progression**	**T Cell phenotype [Reference]**
**Exon 2**	c.227del	p.L76Qfs+53	X	X	X	5 d	IS	Absence of CD4^+^CD127^low^FOXP3^+^ cells, CD4^+^CD127^low^ T cells of carrier express only WT allele (72)
								Increased Th2 differentiation (73)
								Presence of CD25^low^FOXP3^low^ cells, high activation status (74)
			X		X	1 d	died at 8 mo	N/A (75)
	c.232_233del	p.M78Gfs*127	N/A	N/A	N/A	N/A	N/A	FOXP3Δ2 expression upon stimulation (68)
	c.239del	p.A80Dfs*49	N/A	N/A	N/A	N/A	N/A	FOXP3Δ2 expression upon stimulation (68); non-suppressive T cells (76)
	c.303_304del	p.F102Hfs*103	X	X	X	4 mo	HSCT	N/A (77, 78)
	c.305del	p.F102Sfs*27	X	X	X	6 mo	IS	High activation status (79), CD4^+^CD127^low^ T cells of carrier express mostly WT allele
			X			40 y	none	
**Exon 7**	c.736-2A>C	Aberrant splicing	X	X	X	2 mo	IS; died at 8 y	Few CD4^+^FOXP3^+^ cells, increased Th2/Th17-cell differentiation (80)
	c.748_750del	p.K250del	X	X	X	2 mo	IS; HSCT; died at 9 y	N/A (81)
			X	X	X	2 mo	IS	Presence of CD4^+^CD25^+^FOXP3^low^ cells (82)
			X	X		4 mo	IS; HSCT	CD4^+^CD127^low^ T cells of carrier express WT and mutant allele (72)
	c.750_752del c.751_753del	p.E251del p.E251del	X	X	X	N/A	N/A	N/A (3)
			X	X	X	1 mo	IS; HSCT; died at 4 y	N/A (83)
	c.758T>C	p.L253P	X		X	N/A	HSCT	Absence of CD4^+^CD25^+^ cells (84)
	c.767T>C	p.M256T	N/A	N/A	N/A	N/A	N/A	N/A (68)
	c.816+2delT	Aberrant splicing	N/A	N/A	N/A	6 mo	HSCT	N/A (69)
	c.816+2T>A	Aberrant splicing	X	X	X	N/A	HSCT	Absence of CD25^+^CD127^low^ cells, FOXP3Δ7 and FOXP3Δ2Δ7 expression upon stimulation (67)
	c.816+3G>C	Aberrant splicing	N/A	N/A	N/A	N/A	N/A	N/A (68)
	c.816+4A>G	Aberrant splicing	X		X	2 mo	Died at 3 y	N/A (85)
			X	X	X	1 d	IS	Increased Th17-cell differentiation (together with other mutations) (86)
	c.816+5G>A	Aberrant splicing	X		X	6 mo	IS; died at 9 y	FOXP3 expression in CD25^−^ T cells (83)
			X		X	2 mo	IS; HSCT	
			X	X	X	1 d	IS; HSCT	
			X		X	N/A	HSCT	Low FOXP3 expression (84)
			X	X	X	N/A	IS; died at 9 mo	N/A (87)
	c.816+7G>C	Aberrant splicing	X		X	d-mo (6 boys)	Death before 5 y	N/A (85)
			X	X	X	N/A	N/A	N/A (88)

*X, symptoms mentioned in case report; N/A, data not available; IS, immunosuppressive drug treatment; HSCT, hematopoietic stem cell transplantation; d, days; mo, months; y, years*.

## Isoforms Fall Short to Preserve Immune Regulation in IPEX Patients

Frameshift mutations in *FOXP3 exon 2* generate premature stop codons that target mRNAs for nonsense-mediated decay. The rapid mRNA degradation prevents translation into mutated protein, so that only *FOXP3* transcripts lacking exon 2 yield considerable protein expression. Skipping mutations in exon 2 should ensue regular amino acid sequence and expression level of FOXP3Δ2. However, deletion of nucleotide 227 (c.227del) in *FOXP3 exon 2* causes the absence of the CD4^+^CD127^low^FOXP3^+^ T-cell population and only few CD4^+^CD25^+^FOXP3^low^ T cells were found in the periphery (72, 74). Furthermore, due to random X-chromosome inactivation half of the Treg and Tcon cells lack FOXP3-mediated regulation in female mutation carriers. Although IPEX syndrome has not been reported, carriers display a bimodal FOXP3 expression within the CD4^+^CD25^+^CD127^low^ T-cell population for FOXP3 mutations that do not affect Treg-cell development (e.g., c.1150G>A) (72). In contrast, only cells with activated wild-type allele but not IPEX mutations are present among the CD4^+^CD25^+^CD127^low^ T-cell population of subjects carrying the *FOXP3 exon 2* mutation c.227del and a similar trend has been reported for IPEX mutation c.305del (72, 79). Taken together, this suggests that FOXP3Δ2 expression alone is insufficient to promote the generation of Treg cells. Consistent with impaired T-cell homeostasis, T cells from IPEX patients with *FOXP3 exon 2* mutations c.227del and c.305del have a highly activated/differentiated phenotype (73, 74, 79). Noteworthy, FOXP3Δ2 expression is induced in stimulated T cells from IPEX patients with *FOXP3 exon 2* mutations c.232_233del, c.239del and c.305del, albeit individual expression levels differ and appear to be decreased compared to Treg cells from healthy controls (68, 79). The activation and proliferation status of Tcon cells may therefore explain variable FOXP3Δ2 expression patterns for IPEX patients with *FOXP3* mutations c.227del, c.232_233del, c.239del, and c.305del. This notion is corroborated by the recent finding that T cells with enforced expression of *FOXP3 c.239del* via gene editing do not suppress T-cell proliferation (76). Thus, absence of FOXP3fl prevents phenotypical and functional Treg-cell development and FOXP3Δ2 expression alone in IPEX patients with *FOXP3 exon 2* mutations does not characterize *bona fide* Treg cells.

In line with a dominant-negative effect of FOXP3Δ2Δ7 (52), excessive splicing of *FOXP3 exon 7* through IPEX mutations c.736-2A>C, c.816+2delT, c.816+2T>A, c.816+3G>C, c.816+4A>G, c.816+5G>A, and c.816+7G>C decreases FOXP3 expression and abrogates the development of CD4^+^CD25^+^CD127^low^ T cells (67, 80, 84). Moreover, inducible expression of FOXP3 isoforms lacking exon 7 has been reported in TCR-stimulated CD4^+^CD25^−^ T cells from IPEX patients with FOXP3 mutations c.816+2T>A and 816+5G>A (67, 83). Enhanced exclusion of *FOXP3 exon 7* through splice-shifting antisense oligonucleotides negates FOXP3fl/FOXP3Δ2-mediated inhibition of IL-2 and promotes IL-17A expression (55). Consequently, differentiation of Th2 and Th17 cells increase in IPEX patients with mutations in intronic regions that regulate alternative splicing of *FOXP3 exon 7* (80, 86). Only four reports analyzed the phenotype of T cells from IPEX patients with mutations within FOXP3 exon 7. The deletion mutations c.748_750del and c.750_752del/c.751_753del miss amino acid K250 and E251, respectively. Because reading frame and FOXP3 isoform expression ratio is maintained in these mutations, functional properties of this domain could be revealed (65). This is also reflected by the relatively mild T-cell phenotype with reduced FOXP3 expression in preserved CD4^+^CD25^+^ populations (82, 89), and bimodal FOXP3 expression among CD4^+^CD25^+^CD127^low^ T cells in a mutation carrier (72). Similarly the missense mutation *FOXP3 c.758T*>*C*, that generates an amino acid exchange (p.L253P), has no effect on splice variants but prevents the CD4^+^CD25^high^ T-cell phenotype (84). In summary, in-frame mutations of *FOXP3 exon 7* cause dysfunctional FOXP3 isoforms, whereas mutations of intron 6 and 7 cause aberrant splicing that underlines the insufficient T-cell regulation by FOXP3 isoforms lacking exon 7.

## Conclusions

Although activation and proliferation of T cells in IPEX patients may be affected by immunosuppressive drug treatment, important characteristics of FOXP3 isoforms can be deduced: FOXP3fl increases upon TCR stimulation and is indispensable to induce sustainable Treg-related phenotype and functions, whereas FOXP3Δ2Δ7 counteracts the action of FOXP3fl under pro-inflammatory conditions. FOXP3Δ2 does not promote its own expression but supports and may stabilize Treg-mediated immunosuppression. Data from IPEX patients with impaired isoform ratios indicate that modulated *FOXP3* splicing could enhance T-cell responses via exon 7 exclusion (e.g., in tumor-specific T cells) or promote immunosuppression via exon 2 inclusion (e.g., in immunopathology). Essential for this strategy is the targeting of splice-shifting elements that has shown promising potential in other isoform-related diseases (90, 91). Alternatively, also gene editing that enhances FOXP3fl expression may provide a new approach to regulate human T-cell responses (76, 92).

## Author Contributions

The author confirms being the sole contributor of this work and has approved it for publication.

## Conflict of Interest

The author declares that the research was conducted in the absence of any commercial or financial relationships that could be construed as a potential conflict of interest.
